# Disease-Modifying Drugs and Breastfeeding in Multiple Sclerosis: A Narrative Literature Review

**DOI:** 10.3389/fneur.2022.851413

**Published:** 2022-04-15

**Authors:** Fioravante Capone, Angela Albanese, Giorgia Quadri, Vincenzo Di Lazzaro, Emma Falato, Antonio Cortese, Laura De Giglio, Elisabetta Ferraro

**Affiliations:** ^1^Neurology, Neurophysiology and Neurobiology Unit, Department of Medicine, Università Campus Bio-Medico, Rome, Italy; ^2^Merck Serono S.p.A., An Affiliate of Merck KGaA, Rome, Italy; ^3^Multiple Sclerosis Centre, S. Filippo Neri Hospital, Rome, Italy

**Keywords:** multiple sclerosis, pregnancy, breastfeeding, post-partum, disease modifying therapies

## Abstract

Pregnancy-related issues in women with multiple sclerosis (MS) have been receiving increasing attention, with particular interest for the use of disease-modifying therapies (DMTs) before conception, during pregnancy, and postpartum, including breastfeeding. The risk of relapse is higher in the early postpartum period, especially in cases of significant disease activity prior to pregnancy, and thus treatment resumption and/or switching strategies might be necessary. Moreover, breastfeeding provides unmatched health benefits for babies and mothers, and is recommended as the best source of nutrition for infants. Furthermore, a protective role of breastfeeding on MS disease course has not been fully demonstrated and it remains debatable. At the same time, a source of concern is the potential transfer of DMTs into breastmilk and the resulting infant exposure. The use of most DMTs is unlicensed during breastfeeding mainly due to the limited data available on the excretion in human milk and on the effects on infants' exposure. Consequently, women have to face the difficult challenge of choosing between breastfeeding and DMT resumption. The present narrative review summarizes and discusses the available evidence on the safety of DMTs during breastfeeding and the relative approved labels. At the time of diagnosis of MS, specific counseling should be offered to women of childbearing age, making them aware of the possible therapeutic options and their impact on pregnancy and breastfeeding. Women can be encouraged to breastfeed, if clinically feasible, following a review of their medications and clinical status, with a personalized approach.

## Introduction

Multiple sclerosis (MS) is a chronic inflammatory demyelinating disease associated with significant disability (1). A relapsing-remitting pattern is the most common form of the disease (RRMS) and is reported to be present in 72% of patients (2). MS has a definite predominance for women, with a prevalence that is at least three times higher than in men (3). This fact, together with the evidence that MS usually has an onset between the childbearing ages of 20 and 40 years, make issues related to pregnancy of particular concern (4). Indeed, changes in treatment decisions concerning pregnancy may occur following a diagnosis of MS (5).

Pregnancy-related issues in women with MS have been receiving increasing attention in recent years. The most relevant are those relating to the use of disease-modifying therapies (DMTs) before conception, during pregnancy, and postpartum, including breastfeeding. It is now well consolidated that MS has no negative impact on fertility or fetal outcomes (6), likewise, it does not negatively affect disease control or long-term outcomes (6). On the contrary, it is now believed that pregnancy may have benefits in terms of reduced relapse rates. In a meta-analysis of 6,430 pregnancies, annual relapse rates (ARRs) decreased from 0.57 before pregnancy to 0.36, 0.29, and 0.16 during the first, second, and third trimesters, respectively, although a rebound effect was observed post-partum (ARR of 0.85) (7).

The vast majority of DMTs are not licensed for use during pregnancy (8), since their assumption during pregnancy may be associated with the risk of adverse events to the fetus (8). The discontinuation of most DMTs is generally recommended during this period, but this decision should be carefully evaluated on a case-by-case basis by physicians and patients because some DMTs (such as beta interferon) can be continued (9, 10). Since the relapse rate may increase during the post-partum period, there is a clinical dilemma regarding the use of DMTs after pregnancy. In the past, it was held that women who chose to breastfeed should not resume DMT, although a personalized approach is now preferred (11). Breastfeeding is recognized as the best source of nourishment for infants and young children and provides unmatched physical and psychological benefits for infants and mothers (12, 13). WHO recommends exclusive breastfeeding for the first 6 months of life, and breastfeeding continuation—together with safe and adequate complimentary foods—from the age of 6 months for up to 2 years and beyond. There is some evidence to suggest that breastfeeding may reduce ARRs and disability (14). Indeed, some studies have reported that ARRs may remain low for up to 2 years postpartum in women who breastfeed their infants (15). An early meta-analysis found that women who were breastfeeding were around two times less likely to have a post-partum relapse (16). A more recent systematic review and meta-analysis of 24 publications in 2,974 women found that breastfeeding was associated with an odds ratio for post-partum relapses of 0.63 compared to non-breastfeeding women (17). Notwithstanding, there is still some debate on the effectiveness of breastfeeding in reducing relapse rates (18).

In addition, to compound the clinical dilemma, for some DMTs there is also potential harm to the breastfed child. However, at present, regulatory considerations for the use of DMTs during breastfeeding are not the same for all of them and are now being complemented by new evidence, which thus opens new possibilities for their use in clinical practice. The objective of the present narrative review is to summarize the available evidence on the safety of DMTs during breastfeeding and the relative approved labels.

## Methods

We queried PubMed database from inception to October 10, 2021. We included the following search terms: “disease-modifying”, “Interferon β” or “beta interferon”, “glatiramer acetate”, “dimethyl fumarate”, “teriflunomide”, “fingolimod”, “siponimod”, “ozanimod”, “natalizumab”, “cladribine”, “ocrelizumab”, “ofatumumab”, “alemtuzumab” in combination with: “breastfeeding”, “lactation,” or “breastmilk.” The reference lists of the included articles and the relevant links were also manually reviewed for additional eligible articles. We also queried the LactMed database, and we reviewed the reference list for each of the above medications. Studies published in English with full text available were included. The summary of product characteristics was downloaded from official websites of EMA and FDA. The inclusion criteria for this narrative review included the studies regarding the use of DMTs during lactation and the summaries of product characteristics of DMTs approved by EMA and/or FDA.

## DMTs and Breastfeeding

DMTs are generally contraindicated during breastfeeding since they may be transferred into breast milk and result in infant exposure (19). The possible transfer depends on several factors such as its molecular weight, half-life, capacity for protein binding, solubility, metabolism, and relevant active transport mechanisms (20). A drug's volume of distribution must also be considered (21). In general, very large molecules tend to not pass into breast milk. Unfortunately, at present, there is limited information for most DMTs regarding their safety during breastfeeding. The available DMTs are considered individually below.

### Interferon Beta

Interferons are a group of endogenous glycoproteins endowed with immunomodulatory, antiviral and antiproliferative properties. Interferons (IFNs) beta-1a and beta-1b have been first line treatment for MS for decades. The transfer of IFN to breast milk is likely limited by its large molecular weight and the fact that it binds to T cells (21).

A study conducted in 2012 on 6 women receiving 30 μg/week IFN beta-1a found that the highest milk concentrations of IFN beta1a (179 pg/ml) corresponded to a relative infant dose of 0.006% of the maternal dose, strongly suggesting that IFN beta-1a does not significantly transfer into human breast milk (22) and, since the poor oral absorption, it is not likely to reach the bloodstream of the infant (22).

Another study was conducted on 39 infants from women with MS who were breastfeeding while on IFN beta-1a and were followed by the Germanic Multiple Sclerosis and Pregnancy Registry. Most infants included in this study were also exposed during pregnancy and infants were breastfed for an average of 9.2 months (range 1.6–28.5 months) during interferon therapy. No uncommon adverse outcomes were seen during the first year of life and developmental delay, courses of antibiotics and hospitalizations did not differ from the reference German population (23).

According to the LactMed database, none of the mothers receiving IFN beta-1a while breastfeeding has noticed any adverse effects in their breastfed infants (24). Experts' opinion also suggests that IFNs can be used safely by breastfeeding mothers (11). According to the European Medicines Agency (EMA) Summaries of Product Characteristics of IFN beta-1a and beta-1b, no harmful effects on breastfed infants are anticipated, and the products can be used during breastfeeding (25, 26).

Instead, the FDA distinguishes between IFN beta-1a that could be used with caution in nursing women (27, 28) and IFN-beta-1b in which a decision should be made to either discontinue nursing or discontinue the drug, taking into account the importance of drug to the mother (29).

### Glatiramer Acetate

GA, indicated for the treatment of relapsing forms of multiple sclerosis (MS), is a polymer of the aminoacids tyrosine, alanine, glutamate, and lysine with a molecular weight of 5,000–9,000 Daltons (30). Even if there are no data on the transfer of glatiramer acetate into breast milk in humans (30), it is highly unlikely given its large size (21). Additionally, the compound is metabolized into its parent amino acids which pose no risk to the breastfed infant (21). Hellwig et al. reported no noticeable problems in newborns exclusively breastfed for 6 months by three mothers who were receiving glatiramer acetate (31). Another study on nine mothers receiving glatiramer acetate during pregnancy and postpartum who breastfed for 3.6 months on average, no effects were reported in their breastfed infants except for one otherwise normal infant with delayed language development at the 1-year follow-up (32). In an observational study in 34 women who were receiving glatiramer acetate while breastfeeding, no increase in common adverse events was seen (23). Safety data were confirmed for up to 18 months in an extension study including 60 exposed infants and 60 controls (33).

Experts' opinion suggests that GA can be used safely by breastfeeding mothers (11), although according to the EMA Summary of Product Characteristics, a decision must be made whether to discontinue breastfeeding or to discontinue from glatiramer acetate, considering the benefits of breastfeeding for the infant and the benefit of therapy for the mother (30). Also, the FDA label states that it is not known if glatiramer acetate is excreted in human milk. Because many drugs are excreted in human milk, caution should be exercised when glatiramer acetate is administered to a nursing woman (34).

### Dimethyl Fumarate

Dimethyl fumarate is the methyl ester of fumaric acid and it is an oral treatment indicated for the treatment of adult patients with relapsing remitting multiple sclerosis. Dimethyl fumarate is metabolized rapidly with a half-life of about 1 hour and has a large volume of distribution of 60–90 liters for a 240 mg dose (35). While these characteristics would likely indicate that significant transfer of dimethyl fumarate to breast milk does not occur, on the other hand it has a small molecular weight and low protein binding capacity (27–40%) (35), which could indicate significant transfer into milk (11, 21). A recent case study in two lactating women on therapy with dimethyl fumarate calculated the relative infant dosages to be 0.019% and 0.007% of the maternal dosage (36). According to the LactMed database (37), no relevant information has been published so far on the effects in the exposed infants. Given the limited data on the transfer of dimethyl fumarate to breast milk, according to the EMA Summary of Product Characteristics, a risk to infants cannot be excluded accordingly, a decision must be made whether to discontinue breast-feeding or to discontinue dimethyl fumarate therapy, taking into account the benefits of breastfeeding for the infant and the benefit of therapy for the mother (35). In addition, the FDA label states that caution should be exercised when it is administered to a nursing woman (38).

### Teriflunomide

Teriflunomide, the active metabolite of leflunomide, is an immunomodulatory drug inhibiting pyrimidine de novo synthesis indicated for the treatment of adult patients with relapsing remitting multiple sclerosis. Animal studies have documented the transfer of teriflunomide to milk (39). Teriflunomide showed to be teratogenic and embryotoxic in animal studies (39). Moreover, it is eliminated slowly from the plasma, and in the absence of an accelerated elimination procedure, it takes about 8 months to reach plasma concentrations <0.02 mg/l, although due to individual variation in clearance may take up to 2 years (39). According to the LactMed database (40), there is no published experience with teriflunomide during human lactation. Teriflunomide is labeled as contraindicated during lactation by the EMA Summary of Product Characteristics (39). Similarly, the FDA label states that a decision should be made whether to discontinue nursing or to discontinue the drug, taking into account the importance of the drug to the mother (41).

### S1P Inhibitors

Fingolimod is metabolized to the active metabolite fingolimod phosphate, which inhibits the sphingosine-1-phosphate (S1P) receptor (42), and is extensively distributed to body tissues with a volume of distribution of about 1,200 liters with an apparent terminal half-life of 6–9 days (42). The sphingosine 1-phosphate receptor is known to be involved in vascular formation during embryogenesis (32). Fingolimod is an oral treatment indicated for adults and children over 10 years of age with highly active relapsing-remitting multiple sclerosis (MS) (42). Fingolimod showed to be teratogenic in animal studies, where induced fetal loss and organ defects (42). Fingolimod is excreted in milk of treated animals during lactation (32). According to the LactMed database there is no relevant published experience with fingolimod during human breastfeeding. The EMA Summary of Product Characteristics states that women receiving fingolimod should not be breastfeed given the potential for serious adverse reactions in nursing infants (42). The FDA label states that the developmental and health benefits of breastfeeding should be considered along with the mother's clinical need for the drug and any potential adverse effects on the breastfed infant from fingolimod or from the underlying maternal condition (43).

Siponimod and ozanimod have also recently entered the treatment landscape of MS (44, 45). For siponimod, it is unknown whether the compound or its major metabolites are excreted in human milk, although they are excreted in the milk of rats (44). Ozanimod and its metabolites are excreted in milk of treated animals during lactation and pose a potential for serious adverse reactions in nursing infants (34). For both treatments there is no published experience of their use in nursing women. The EMA Summaries of Product Characteristics state that siponimod (44) should not be used during breastfeeding and that women receiving ozanimod (45) should not breastfeed, respectively. Similarly, for fingolimod the FDA labels state that their use during breastfeeding should take into account the mother's clinical need for the drug and any potential adverse effects on the breastfed infant (46, 47).

### Natalizumab

Natalizumab is a recombinant humanized anti-α4-integrin antibody (48), an infusion treatment indicated in adults with highly active relapsing remitting multiple sclerosis (RRMS). It has been detected in low amounts in breast milk of nursing women (49). Proschmann et al. reported that in the majority of the mother–infant serum pairs (6/11) and in all breast milk samples, free Natalizumab was detectable with a significant association with the time since the last infusion (49). According to the conventional model of drug passage into breast milk, large molecules such as maternal immunoglobulins can pass into colostrum because of the wide spaces between mammary epithelial cells (49).

In a case-report of a lactating woman with MS who was taking natalizumab while breastfeeding, the mean concentration of natalizumab in breast milk was 0.93 μg/ml/day, with a relative infant dose of 1.74% of the maternal dose (50). Moreover, transfer of natalizumab into human milk was seen to increase with time and subsequent injections; the highest concentration (2.83 μg/ml) was seen at day 50 with a relative infant dose of 5.3% (50).

Ciplea et al. recently reported on 17 patients from the German Multiple Sclerosis and Pregnancy Registry (DMSKW) who received natalizumab during lactation (51). No negative impact was observed on infant health or development that was attributable to breast milk exposure after a median follow-up of 1 year (51). Infants exposed to natalizumab during the third trimester had a lower birth weight and more hospitalizations in the first year of life (51). However, the concentration of natalizumab in breast milk and serum of infants was low (51). According to the EMA Summary of Product Characteristics, since the effect of natalizumab on newborn/infants is unknown, breastfeeding should be discontinued during treatment with natalizumab (48). The FDA product label does not contain specific contraindications during breastfeeding, and only reports that natalizumab has been detected in human milk and that the effects of this exposure are unknown (52).

### Cladribine Tablets

Cladribine tablets, an oral DMT indicated for the treatment of highly active RRMS patients, is a chlorinated analog of deoxyadenosine that is relatively resistant to ADA-mediated deamination (53). Therefore, cladribine accumulates in lymphocytes and depletes them through apoptosis (53). Evidence from the cladribine clinical development program were generally consistent with epidemiological data on pregnancy outcomes for the general population or women with multiple sclerosis (54). There were showed no congenital malformations in pregnancies that occurred during cladribine treatment or within 6 months after the last dose (54). Furthermore, a non-interventional post-authorization safety study has been initiated to obtain more information (54).

To date, there are only very limited data on the transfer of cladribine into breast milk. A recent case study documented the transfer of cladribine into human milk in a woman with MS who was breastfeeding (55). The patient experienced a relapse at 4 months postpartum and began therapy with 20 mg cladribine; although she discontinued breastfeeding, she donated milk samples for study. Cladribine levels in milk were 281.2 ng/mL at 1 hour following a 20-mg dose, with a rapid decline in milk concentration over a period of 12–24 h. The levels were undetectable in the milk samples collected after administration of her last dose at 48, 72, and 96 h (55).

In the EMA label, cladribine is contraindicated during breastfeeding and for a period of one week following the last dose (53). Similarly, the FDA contraindicates breastfeeding during cladribine treatment and until 10 days after the last dose (56).

### Anti-CD20 Therapies

Two anti-CD20 therapies have already received EMA and FDA approval for the treatment of MS: Ocrelizumab and Ofatumumab.

Ocrelizumab is a recombinant humanized anti-CD20 monoclonal antibody (57). It is unknown whether ocrelizumab or its metabolites are transferred in human milk, and the available pharmacodynamic/toxicological data in animals have shown excretion of ocrelizumab in milk (57). Given its large molecular weight, the amount of ocrelizumab transferred in milk is likely to be low and would likely be degraded in the infant's gastrointestinal tract. Considering that most monoclonal antibodies used in MS are either for induction or have a prolonged dose interval, this will undoubtedly limit the infant's exposure (20). Only a few infants have been exposed to ocrelizumab during breastfeeding with no evidence of harm (58). Ciplea et al. recently reported on 6 patients from the DMSKW (German MS and Pregnancy Registry) who received anti-CD20 antibodies (3 rituximab, 2 ocrelizumab, and 1 rituximab and ocrelizumab) during lactation (51). Low levels of transfer into breast milk were detected. Notwithstanding, whether these minimally detectable breast milk levels pose any risk to the infants is unknown, leading many experts to be cautious (51). According to the EMA Summary of Product Characteristics, women should be advised to discontinue breastfeeding during therapy with ocrelizumab (57). The FDA label states that the developmental and health benefits of breastfeeding should be considered along with the mother's clinical need for the drug and any potential adverse effects on the breastfed infant from the drug or from the underlying maternal condition (59).

Ofatumumab is a CD20-directed cytolytic antibody that is approved by the FDA and EMA for the treatment of RRMS (60). According to LactMed database (61) there are currently no data about the secretion of ofatumumab in human milk or its effects on breastfed infants, in particular, about the potential B-cell depletion The EMA label states that the use of ofatumumab in women during lactation has not been studied. Since, the excretion of IgG antibodies in human milk occurs during the first few days after birth, which is decreasing to low concentrations soon afterwards. Consequently, a risk to the breast-fed child cannot be excluded during this short period. Afterwards, ofatumumab could be used during breastfeeding, if clinically needed. If the patient was treated with ofatumumab up to the last few months of pregnancy, breast-feeding can be started immediately after birth (62). The FDA label states that the developmental and health benefits of breastfeeding should be considered along with the mother's clinical need for ofatumumab and any potential adverse effects on the breastfed infant from ofatumumab or from the underlying maternal condition (63).

### Alemtuzumab

Alemtuzumab is a humanized IgG1 kappa monoclonal antibody specific for CD52 (64), indicated for adults with highly active relapsing remitting multiple sclerosis. Alemtuzumab has been detected in the milk and offspring of lactating female mice, but no information is available on the use of alemtuzumab during breastfeeding in humans (64). As with other monoclonal antibodies, considering its large molecular weight it is unlikely to be transferred to breast milk in amounts that are clinically significant. The EMA Summary of Product Characteristics specifies that breastfeeding should be discontinued during each course of treatment with alemtuzumab and for 4 months following the last infusion of each treatment course (64). However, it is also noted that the benefits of conferred immunity through breast milk may outweigh the risks of potential exposure to alemtuzumab for the suckling newborn/infant (64). The FDA label states that, because of the potential for serious adverse reactions in nursing infants from the drug, a decision should be made whether to discontinue nursing or to discontinue the drug, considering the importance of the drug to the mother (65).

### BTK Inhibitors

Three inhibitors of Bruton's tyrosine kinase (BTK) are currently in development for treatment of MS (66). BTK is responsible for signal transmission through several receptors in B cells and myeloid cells and is thus a valid target in MS (67). Evobrutinib has shown promising results in a Phase 2 study (67) and is currently being investigated in a Phase 3 trial (68). There is still no information on the transfer of evobrutinib into breast milk, and women who are pregnant or breastfeeding have been excluded from clinical trials.

## Discussion

At present, there is limited information for most DMTs regarding human milk transfer and effects on the breastfed infant. Consequently, very few DMTs are explicitly licensed for use during breastfeeding and a case-by-case decision is recommended for most of them. Animal and clinical studies about the potential transfer of DMTs in the breast milk supported the recommendations from EMA and FDA regulatory authorities, which do not always overlap each other (Table 1). Furthermore, case reports and post-marketing data are expanding our knowledge about the safety of DMTs' use in nursing woman.

**Table 1:** DMDs: EMA and FDA SmPCs focusing on breastfeeding indication.

	**EMA approved SmPCs**	**FDA approved SmPCs**
**Interferon** **β-1a im (AVONEX**^®^**)**	Limited information available on the transfer of interferon β 1a into breast milk, together with the chemical/physiological characteristics of interferon β, suggests that levels of interferon β-1a excreted in human milk are negligible. No harmful effects on the breastfed newborn/infant are anticipated. Avonex can be used during breast-feeding.	It is not known whether AVONEX is excreted in human milk.
**Interferon** **β-1a sc (REBIF**^®^**)**	Limited information available on the transfer of interferon β 1a into breast milk, together with the chemical/physiological characteristics of interferon β, suggests that levels of interferon β-1a excreted in human milk are negligible. No harmful effects on the breastfed newborn/infant are anticipated. Rebif can be used during breast-feeding.	It is not known whether REBIF is excreted in human milk. Because many drugs are excreted in human milk, caution should be exercised when REBIF is administered to a nursing woman.
**PEG Interferon** **β-1a sc (PLEGRIDY**^®^**)**	It is not known whether peginterferon β-1a is secreted in human milk. Limited information available on the transfer of interferon β-1a into breast milk, together with the chemical/physiological characteristics of interferon β, suggests that levels of interferon β-1a excreted in human milk are negligible. No harmful effects on the breastfed newborn/infant are anticipated. Peginterferon β-1a can be used during breast-feeding.	It is not known whether this drug is excreted in human milk. Because many drugs are excreted in human milk, caution should be exercised when PLEGRIDY is administered to a nursing woman.
**Interferon** **β- 1b** **(BETAFERON/BETASERON**^®^**)**	Limited information available on the transfer of interferon β 1b into breast milk, together with the chemical/physiological characteristics of interferon beta, suggests that levels of interferon β−1b excreted in human milk are negligible. No harmful effects on the breastfed newborn/infant are anticipated. Betaferon can be used during breast-feeding.	It is not known whether BETASERON is excreted in human milk. Because many drugs are excreted in human milk and because of the potential for serious adverse reactions in nursing infants from BETASERON, a decision should be made to either discontinue nursing or discontinue the drug, taking into account the importance of drug to the mother.
**Interferon** **β-1b (EXTAVIA**^®^**)**	Limited information available on the transfer of interferon β-1b into breast milk, together with the chemical/physiological characteristics of interferon beta, suggests that levels of interferon β−1b excreted in human milk are negligible. No harmful effects on the breast-fed newborn/infant are anticipated. Extavia can be used during breast-feeding.	It is not known whether interferon β-1b is excreted in human milk. Because many drugs are excreted in human milk and because of the potential for serious adverse reactions in nursing infants from interferon β-1b, a decision should be made to either discontinue nursing or discontinue the drug, taking into account the importance of drug to the mother.
**Glatiramer acetate (COPAXONE** ^®^ **)**	It is unknown whether dimethyl fumarate or its metabolites are excreted in human milk. A risk to the newborns/infants cannot be excluded. A decision must be made whether to discontinue breast-feeding or to discontinue Copaxone therapy. The benefit of breast-feeding for the child and the benefit of therapy for the woman should be taken into account.	It is not known if glatiramer acetate is excreted in human milk. Because many drugs are excreted in human milk, caution should be exercised when COPAXONE is administered to a nursing woman.
**Dimethyl fumarate (TECFIDERA** ^®^ **)**	It is unknown whether dimethyl fumarate or its metabolites are excreted in human milk. A risk to the newborns/infants cannot be excluded. A decision must be made whether to discontinue breast-feeding or to discontinue Tecfidera therapy. The benefit of breast-feeding for the child and the benefit of therapy for the woman should be taken into account.	It is not known whether this drug is excreted in human milk. Because many drugs are excreted in human milk, caution should be exercised when TECFIDERA is administered to a nursing woman.
**Teriflunomide (AUBAGIO** ^®^ **)**	Animal studies have shown excretion of teriflunomide in milk. Teriflunomide is contraindicated during breast-feeding.	It is not known whether this drug is excreted in human milk. Because many drugs are excreted in human milk and because of the potential for serious adverse reactions in nursing infants from AUBAGIO a decision should be made whether to discontinue nursing or to discontinue the drug, taking into account the importance of the drug to the mother.
**Fingolimod (GILENYA** ^®^ **)**	Fingolimod is excreted in milk of treated animals during lactation. Due to the potential for serious adverse reactions to fingolimod in nursing infants, women receiving Gilenya should not breastfeed.	There are no data on the presence of fingolimod in human milk, the effects on the breastfed infant, or the effects of the drug on milk production. Fingolimod is excreted in the milk of treated rats. The developmental and health benefits of breastfeeding should be considered along with the mother's clincial need for GILENYA and any potential adverse effects on the breastfed infant from GILENYA or from the underlying maternal condition.
**Siponimod (MAYZENT** ^®^ **)**	It is unknown whether siponimod or its major metabolites are excreted in human milk. Siponimod and its metabolites are excreted in the milk of rats. Siponimod should not be used during breast-feeding.	The developmental and health benefits of breastfeeding should be considered along with the mother's clinical need for MAYZENT and any potential adverse effects on the breastfed infant from MAYZENT or from the underlying maternal condition.
**Ozanimod (ZEPOSIA** ^®^ **)**	Ozanimod/metabolites are excreted in milk of treated animals during lactation. Due to the potential for serious adverse reactions to ozanimod/metabolites in nursing infants, women receiving ozanimod should not breastfeed.	The developmental and health benefits of breastfeeding should be considered along with the mother's clinical need for ZEPOSIA and any potential adverse effects on the breastfed infant from ZEPOSIA or from the underlying maternal condition.
**Natalizumab (TYSABRI** ^®^ **)**	Natalizumab is excreted in human milk. The effect of natalizumab on newborn/infants is unknown. Breast-feeding should be discontinued during treatment with natalizumab.	TYSABRI has been detected in human milk. The effects of this exposure on infants are unknown.
**Cladribine (MAVECLAND** ^®^ **)**	It is not known whether cladribine is excreted in human milk. Because of the potential for serious adverse reactions in breast-fed infants, breast-feeding is contraindicated during treatment with MAVENCLAD and for 1 week after the last dose.	MAVENCLAD is contraindicated in breastfeeding women because of the potential for serious adverse reactions in breastfed infants. Advise women not to breastfeed during dosing with MAVENCLAD and for 10 days after the last dose. There are no data on the presence of cladribine in human milk, the effects on the breastfed infant, or the effects of the drug on milk production.
**Ocrelizumab (OCREVUS** ^®^ **)**	It is unknown whether ocrelizumab/metabolites are excreted in human milk. Available pharmacodynamic/toxicological data in animals have shown excretion of ocrelizumab in milk. A risk to neonates and infants cannot be excluded. Women should be advised to discontinue breast-feeding during Ocrevus therapy.	The developmental and health benefits of breastfeeding should be considered along with the mother's clinical need for OCREVUS and any potential adverse effects on the breastfed infant from OCREVUS or from the underlying maternal condition.
**Ofatumumab (KESIMPTA** ^®^ **)**	The use of ofatumumab in women during lactation has not been studied. It is unknown whether ofatumumab is excreted in human milk. In humans, excretion of IgG antibodies in milk occurs during the first few days after birth, which is decreasing to low concentrations soon afterwards. Consequently, a risk to the breast-fed child cannot be excluded during this short period. Afterwards, ofatumumab could be used during breast-feeding if clinically needed. However, if the patient was treated with ofatumumab up to the last few months of pregnancy, breast-feeding can be started immediately after birth.	There are no data on the presence of ofatumumab in human milk, the effects on the breastfed infant, or the effects of the drug on milk production. Human IgG is excreted in human milk, and the potential for absorption of ofatumumab to lead to B-cell depletion in the infant is unknown. The developmental and health benefits of breastfeeding should be considered along with the mother's clinical need for KESIMPTA and any potential adverse effects on the breastfed infant from KESIMPTA or from the underlying maternal condition.
**Alemtuzumab (LEMTRADA** ^®^ **)**	Alemtuzumab was detected in the milk and offspring of lactating female mice. It is unknown whether alemtuzumab is excreted in human milk. A risk to the suckling newborn/infant cannot be excluded. Therefore, breast-feeding should be discontinued during each course of treatment with LEMTRADA and for 4 months following the last infusion of each treatment course. However, benefits of conferred immunity through breast milk may outweigh the risks of potential exposure to alemtuzumab for the suckling newborn/infant.	because of the potential for serious adverse reactions in nursing infants from LEMTRADA, a decision should be made whether to discontinue nursing or to discontinue the drug, taking into account the importance of the drug to the mother.

*DMDs, disesase modifying drugs; EMA, european medicine agency; FDA, food and drug administration; SmPCs, summary of product characteristics*.

In MS, clinical practice and expert opinions suggest that first-line injectable medications such as IFN-beta and GA can be used during breastfeeding (23, 31–33) while there are limited data and no guidelines for monoclonal antibodies use during breastfeeding (17). For monoclonal antibodies, recent efforts have been made to bridge the gap in safety data during breastfeeding due to their widespread use in diseases other than MS. In fact, the 2018 American College of Rheumatology guidelines discussed the use of biologics while breastfeeding (69) and the 2019 American Gastrointestinal Association recommendations supported their use in this phase (70).

The therapeutic options for MS have expanded, and post-marketing data from breastfeeding women with MS are being reported. Many authors are optimistic that most women with MS can have children and that they could be encouraged to breastfeed (71). The unmatchable benefits coming from breastfeeding for the physical and psychological health of the mother and the infant are globally recognized.

The impact that breastfeeding may have on relapses is also an area of significant debate. The meta-analysis by Krysko et al. indicates that breastfeeding is somewhat protective against relapses in the postpartum phase (17) and there is also some evidence suggesting that the impact is most evident in women who exclusively breastfeed (72). Nonetheless, it is still unknown if the reduced risk is cumulative with the protection conferred by early restart of DMTs. Therefore, gathering additional information on the impact of the available DMTs on breastfeeding is mandatory to properly counsel and treat women with MS in the post-partum period.

## Conclusions

Breastfeeding provides unmatched physical and psychological benefits for infants and mothers (12, 13). Increasing evidence shows that foregoing breastfeeding is not necessary in the vast majority of women with MS, and exclusive breastfeeding can be encouraged, if desired by the mother, on a case-by-case basis, particularly in women with milder disease (73). Proper counseling and treatment selection should be considered at diagnosis of MS in women of childbearing age who desire children, possibly avoiding, if the clinical conditions allow to do so, drugs that have teratogenic potential, and drugs that may be linked to severe relapses following cessation, such as natalizumab or fingolimod (73). Other treatments, such as cladribine tablets and alemtuzumab, could be considered because even though they are not indicated during pregnancy and breastfeeding, they offer time windows for family planning due to the intermittent posology which guarantee disease control also during drug-free periods.

Resuming DMTs after delivery to prevent early postpartum relapses is a matter of debate and no guidelines currently exist. To help the clinician in providing a recommendation for any treatment resumption after delivery, it seems reasonable to check for disease activity shortly after pregnancy, with a brain and spine MRI. The management should be discussed with the mother, with a risk-based stratification approach (73). For patients at “low” risk of reactivation in the post-partum (without relapses in the year before pregnancy and during pregnancy and no disease activity at post-partum MRI), exclusive breastfeeding could be encouraged for at least 6 months, considering restarting of a DMT later when breastmilk is no longer the baby's primary source of nutrition. In these cases, interferons (according to the EMA Summaries of Product Characteristics) or GA (according to experts' opinions) could be used during lactation. Patients at “intermediate” risk of reactivation in the post-partum (e.g., subclinical disease activity at post- partum MRI, single mild relapse in the year before pregnancy), could be encouraged to resume, immediately after delivery, DMTs such as interferons (according to the EMA Summaries of Product Characteristics) or GA (according to experts' opinions) while lactating.

In patients at “high” risk of reactivation in the post-partum (e.g., multiple relapses in the pre-pregnancy year, relapses during pregnancy), it would be advisable to restart a highly effective DMT in the immediate post-partum. For women who prefer to breastfeed, monoclonal antibodies could be considered, due to their rapidity of action and their molecular properties that make unlikely their passage in breast milk and into the baby's circulation. However, it should be considered that monoclonal antibodies are not currently approved by regulatory agencies for use during lactation, with the exception of ofatumumab that, according to EMA label, could be used during breastfeeding, if clinically needed.

In general, the decision must always be assessed on a case-by-case basis, adequately informing patients, and listening to their wishes. Further efforts are required to expand knowledge of treatment-related issues during breastfeeding. Post-marketing clinical registries and prospective clinical trials would be particularly helpful in achieving this goal.

## Author Contributions

FC: conceptualization and original draft preparation. AA and GQ: conceptualization and reviewing. VD and EFe: conceptualization, supervision, and reviewing. EFa, AC, and LD: methodology and reviewing. All authors contributed to the article and approved the submitted version.

## Funding

This work has been funded by Merck Serono S.p.A., Rome, Italy, An Affiliate of Merck.

## Conflict of Interest

FC has received travel grants from Biogen, Merck, Sanofi-Genzyme, and Roche and research grants from Merck. EFe has received travel grants from Biogen, Merck, Sanofi-Genzyme, Novartis and Roche. AA and GQ are employees of Merck Serono S.p.A., Rome, Italy, An Affiliate of Merck KGaA, Darmstadt, Germany. The authors declare that this study received funding from Merck Serono S.p.A., Rome, Italy, an Affiliate of Merck. The funder had the following involvement in the study: AA and GQ are employees of Merck and as authors were involved in the manuscript ideation, drafting and finalization. The reviewer EP declared a past co-authorship with one of the authors LD to the handling Editor.

## Publisher's Note

All claims expressed in this article are solely those of the authors and do not necessarily represent those of their affiliated organizations, or those of the publisher, the editors and the reviewers. Any product that may be evaluated in this article, or claim that may be made by its manufacturer, is not guaranteed or endorsed by the publisher.
